# Catalytic Ethylene Oligomerization over Imine-Linked Covalent-Organic Frameworks with Coordinative Ni(II) and Cr(III)

**DOI:** 10.1021/acssuschemeng.5c01284

**Published:** 2025-05-09

**Authors:** Lijun Guo, Yingchuan Zhang, Wei Chen, Yang Li, Da Song, Weiwei Yang, Jin Huang, Feng Li, Cuiqin Li, Zhengxiao Guo

**Affiliations:** † Provincial Key Laboratory of Polyolefin New Materials, College of Chemistry & Chemical Engineering, 117792 Northeast Petroleum University, Daqing 163318, PR China; ‡ Department of Chemistry, 25809 The University of Hong Kong, Hong Kong SAR, 99907, PR China; § Guangzhou Institute of Energy Conversion, Chinese Academy of Sciences, Guangzhou 510640, PR China

**Keywords:** covalent-organic framework, ethylene oligomerization, Box–Behnken design, heterogeneous catalysis, cyclic stability

## Abstract

Covalent-organic frameworks (COFs) hold great promise for heterogeneous catalysis due to their porous structure for gas adsorption and tunable functionality for bond activation. This study reports two flower-like shaped imine-linked COFs with coordinative Ni­(II) and Cr­(III) species, to endow Ni/PD-TPA COF and Cr/PD-TPA COF, for catalytic ethylene oligomerization. The synergy between imine nitrogen atoms and coordinated metals enables an exceptional activity (16.70 × 10^5^ and 19.90 × 10^5^ g/(mol M·h)) with high selectivity of butene and hexane (∼98.5%) in long-span catalytic processes, respectively. To optimize multiple reaction parameters (e.g., cocatalyst dosage, temperature, and time), Box–Behnken design (BBD) is employed as a statistical tool to establish a precise model integrating process parameters, catalyst structures, and catalytic performance. The optimized models exhibit high precision (indicated by high *R* values) in predicting the activity and selectivity of ethylene oligomerization over Cr/PD-TPA COF and Ni/PD-TPA COF, indicating the promise of BBD in reaction engineering and process design. M/PD-TPA COFs are highly active after three recycles and deliver the highest activity at ambient temperature and low pressure with a minimal quantity of cocatalysts compared with other catalysts. These findings demonstrate the potential of metal-loaded COFs for sustainable heterogeneous catalysis and provide insights into the optimization of reaction parameters using statistical methods.

## Graphical abstract

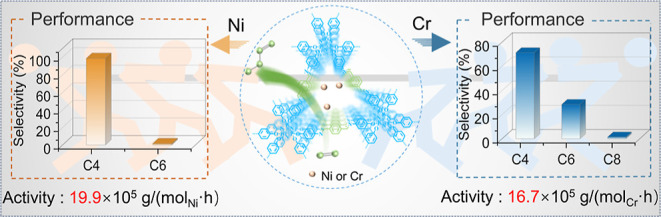

## Introduction

α-Olefins (C_*n*_H_2*n*_) are basic chemicals that serve as the key building blocks and intermediates for various fuels and consumer products such as linear low-density polyethylene (C_4_–C_8_), plasticizers (C_6_–C_10_), synthetic lubricants (C_8_–C_18_), and detergents (C_10_–C_18_).^1–3^ Traditionally, α-olefins are primarily obtained through wax cracking processes, petrochemical extraction, and ethylene oligomerization. However, thermal cracking has been gradually phased out due to the scarcity of wax sources, high energy consumption, and suboptimal quality of the α-olefin products. Ethylene oligomerization has gained increased attention due to its flexibility in terms of feedstocks (e.g., ethylene), oligomerization processes, and narrow-range distribution of final products.^4^ Until recently, this approach accounts for 94.1% of total α-olefins production, but an efficient catalytic system is still desirable toward efficient and clean industrialization of ethylene oligomerization.

Over the past decades, a variety of transition-metal-based homogeneous catalysts have been reported for selective ethylene oligomerization. However, these catalysts suffer from low durability, high sensitivity to impurities, and poor recyclability from the product stream, which are main issues limiting the sustainable conversion of ethylene to oligomers.^5^ In contrast, heterogeneous catalysts have emerged as ideal alternatives with relatively higher recyclability and structural tunability.^6^ Various framework catalysts have been developed, including zeolites (e.g., ZSM-5, MCM),^7,8^ alumina framework,^9–11^ and metal–organic frameworks (MOFs).^12–14^ In particular, MOFs provide unique porous structures for controlled metal loading and organic functionalization.^5,17^ For example, a MOF-supported (bpy)­Ni^II^ moiety (NU-1000-(bpy)­Ni^II^) shows a yield of >95% for ethylene dimerization,^15^ and a Ni-doped MOF (Ni-MOF-5) with methylaluminoxane (MAO) as a cocatalyst exhibits a high activity of 9040 g/(g_catalyst_·h) under relatively mild conditions (35 °C, 50 bar).^18^

In comparison, covalent-organic frameworks (COFs) have rarely been employed in catalytic ethylene oligomerization. As crystalline porous organic polymers with predesignated structures and tunable functionalities,^19^ COFs show relatively higher flexibility and environmental beingness for various applications such as chemical sensors,^20–22^ mass transport,^23,24^ adsorption and separation,^25,26^ and catalysis.^27–29^ In heterogeneous reactions, the tunable nanopores of COFs can provide a confined space, making them as ideal nanoreactors for efficient ethylene adsorption and oligomerization. Moreover, the confinement of porous structures can be further mediated by linkage and pore-surface engineering, which further influences the catalytic activity and product selectivity of the COF catalysts. The first demonstration of COF as catalysts for ethylene oligomerization, however, is reported very recently, where triazine porous aromatic frameworks (CTFs) with coordinative Ni­(II) species enable selective production of C_6+_ olefins.^30^ Li et al. reported two Schiff-base COFs with butanedione and *p*-benzaldehyde as subconstruction units, which are then coordinated with Ni species.^31^ These COFs exhibit good catalytic performance in ethylene oligomerization upon activation by the cocatalyst MAO. However, the understanding of linkage and pore-surface engineering is still insufficient since most previous results involve only single-factor evaluation for the effect of COF linkers on ethylene oligomerization performance.^16^

Since there are multiple variables in terms of COF structures and reactions conditions, statistical tools can be used to reveal the relationships between the performance and numerous variables toward efficient ethylene oligomerization. For example, Soheili et al. evaluated the effect of various parameters (ligand type, metal ratio, reaction temperature, pressure) on C_6_ selectivity and productivity catalyzed by chromium complexes via central composite design (CCD) as an important tool of response surface methodology (RSM). This approach precisely summarizes the high-throughput results as amine-based complexes have higher activities for ethylene trimerization but lower thermal stability than those of pyridine-based complexes.^32^ Box–Behnken design (BBD) is also a type of RSM for specifically evaluating nonlinear relationships between indexes and factors and is more efficient than CCD because of fewer runs in a three-factor experimental design and particularly useful by avoiding extreme treatment combinations.^33^

Here, an imine-linked COF is synthesized from polyamino blocks (PD-(NH_2_)_6_) and 1,4-phthalaldehydes (TPA),^34^ and Ni­(II) and Cr­(III) species are cooperatively loaded in the framework to endow Ni/PD-TPA COF and Cr/PD-TPA COF for catalytic ethylene oligomerization (Scheme 1a). Chromium­(III)- and nickel­(II)-based complexes are widely recognized as effective catalysts for the production of higher olefins, with nickel predominantly facilitating ethylene dimerization, while chromium exhibits superior performance in ethylene trimerization and tetramerization.^35^ These catalytic differences are mediated by distinct electronic structures and mechanistic pathways. Despite some progress in COF-based catalysis in ethylene oligomerization, research has largely focused on nickel as the active center, with limited exploration of chromium-based systems. This study seeks to provide deeper insights into the catalytic behaviors and mechanistic distinctions of these metal centers within the COF. These COF catalysts are extensively characterized by structural, morphological, and thermal analyses. The crucial parameters in terms of reaction conditions and structural properties of COF catalysts are statistically associated via single-factor experiment and BBD toward optimal conditions for ethylene oligomerization (Scheme 1b). These complementary tools rapidly predict the correlation between the variables and the response variables with high precision. Moreover, a narrow product distribution is obtained by highly active coordination controlled oligomerization of M/PD-TPA COFs (Scheme 1c). Recycle experiments and comparisons with other supported catalyst-based performances and processes demonstrated the advantages of M/PD-TPA COFs in environmental acceptability, economic viability, and recyclability.

**Scheme 1:**
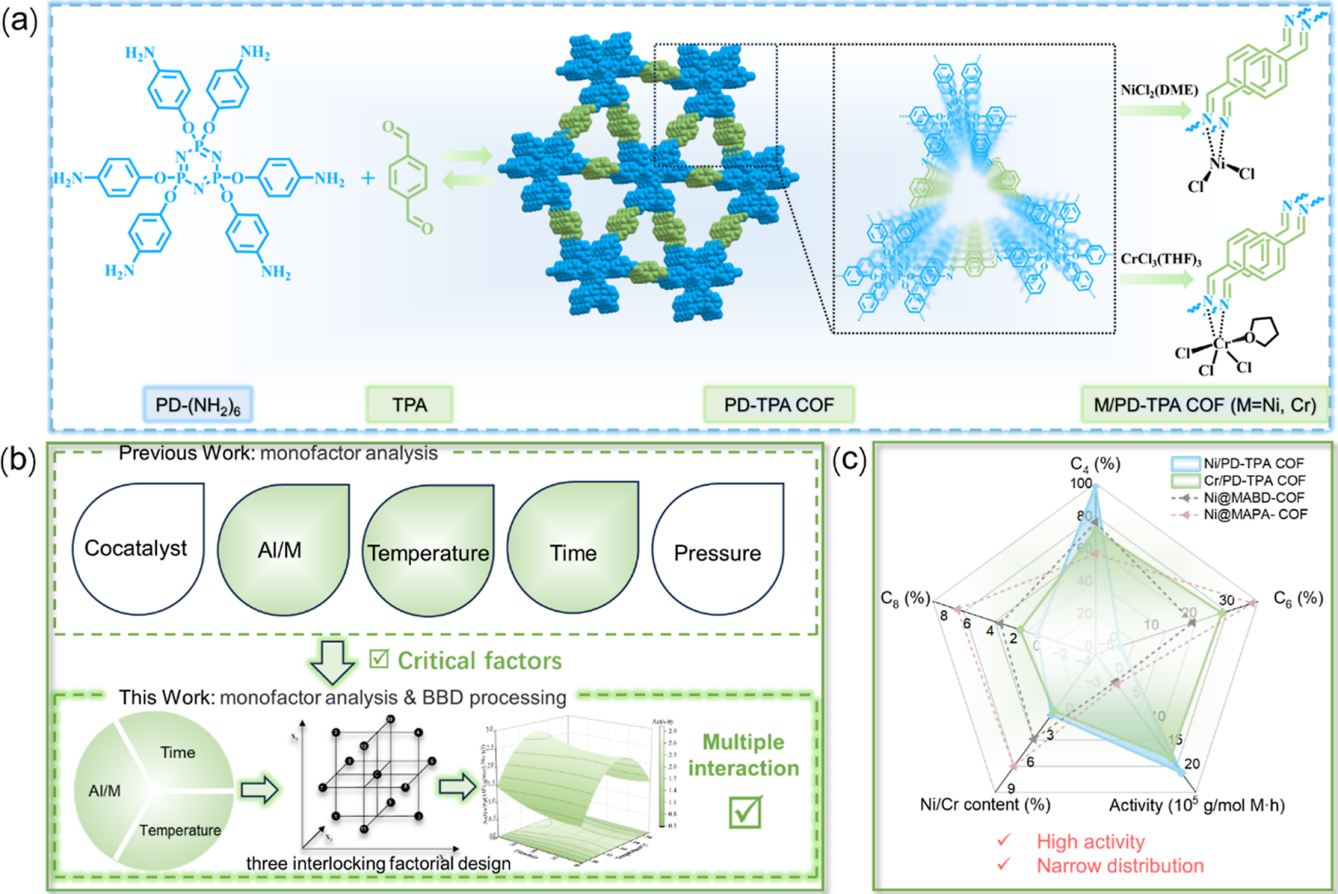
(a) Synthesis of PD-TPA COF and M/PD-TPA COFs (M = Ni, Cr), (b) Schematic Illustration of the BBD Process to Optimize Reaction Conditions of Ethylene Oligomerization, and (c) Comparison of the Catalytic Performance Obtained in This Work and Literature^31^

## Experimental Section

### Synthesis of M/PD-TPA COFs (M = Ni, Cr)

PD-(NH_2_)_6_ (0.1176 g, 0.15 mmol), TPA (0.0604 g, 0.45 mmol), and acetic acid (0.1 mL) were added into a toluene-dioxane binary solvent (v/v: 2/1, 15 mL) in the solvothermal reactor under a nitrogen atmosphere. The mixture was then heated at 120 °C for 72 h under a nitrogen atmosphere. After being cooled to room temperature, the precipitate was collected by filtration and washed with deionized water and 1,4-dioxane three times, respectively. Finally, the solid was dried in a vacuum oven at 120 °C for 12 h to obtain the final product (PD-TPA COF) with an overall yield of 95%. Solid-state ^13^C NMR (100 MHz, ppm): δ 122, 136, 144, 149, 153.

PD-TPA COF (0.1078 g, 0.1 mmol) was dispersed in 25 mL of methylene chloride by ultrasonication under (DME)­NiCl_2_ (0.1318 g, 0.6 mmol) in ethanol (10 mL) was then added, and the reaction mixture was stirred at room temperature for 24 h under nitrogen atmosphere to obtain a brown precipitate. The precipitate was collected by filtration, washed three times with ethanol to remove the unreacted [(DME)­NiCl_2_], and dried at 65 °C for 12 h in the vacuum oven to afford Ni/PD-TPA COF as a brown solid with a yield of 87% (atomic percentage of Ni = 1.81, determined by ICP). Similarly, Cr/PD-TPA COF was synthesized from PD-TPA COF (0.1078 g, 0.1 mmol) and CrCl_3_(THF)_3_ (0.2086 g, 0.6 mmol) in THF by following the same procedure. Cr/PD-TPA COF was isolated as a brown powder with a yield of 75% (atomic percentage of Cr = 1.39).

## Results and Discussion

### Synthesis and Characterizations

PD-TPA COF is synthesized via the Schiff-base reaction under solvothermal conditions.^36^ PD-(NH_2_)_6_ is selected as the node due to its unique flexibility, and TPA serves as the linker. Given the high porosity and large pore size of imine-linked COFs as well as the rich chelating sites, Ni­(II) and Cr­(III) are further loaded to render M/PD-TPA COFs (M = Ni or Cr). As shown in Figure S1a, FTIR spectra confirm the successful synthesis of PD-TPA COF and M/PD-TPA COFs. A characteristic peak at 1619 cm^–1^ is assigned to the stretching of C=N bonds (υ_C=N_),^37^ and the absence of C=O bands around 1700 cm^–1^ for TPA further indicates the occurrence of Schiff-base condensation. Interestingly, a slight blue-shift in the υ_C=N_ from 1619 to 1623 cm^–1^ is observed in M/PD-TPA COFs, which can be attributed to the strengthening of the C=N bonds due to the coordination between the PD-TPA COFs and metal ions. ^13^C SSNMR spectrum of PD-TPA COF (Figure S1b) confirms the existence of imine carbon (154 ppm) and phenyl carbons (121, 136, 145, 149 ppm).^38^

The XPS spectra of M/PD-TPA COFs are shown in Figure S1c,d. Peaks at 284.8, 285.8, and 288.8 eV in C 1s spectra can be assigned to C=C/C–C, C=N bonds, and π–π conjugation, respectively.^39,40^ The existence of imine bonds is further confirmed by N 1s spectra, in which two N 1s core-level peaks are observed at 397.9 and 399.4 eV that are associated with N=P and N=C bonds,^41^ respectively. The Ni 2p spectrum shows double characteristic peaks located at 855.9 and 873.2 eV and can be assigned to Ni 2p_3/2_ and Ni 2p_3/1_, respectively,^42^ suggesting the successful coordination of Ni^2+^ in PD-TPA COF. Ni/PD-TPA COF exhibits a similar pattern in C 1s and N 1s spectra but with Cr 2p_3/2_ and Cr 2p_1/2_ peaks at 577.5 and 587.3 eV, indicating the coordination of Cr^3+^ species.^8,43^

The TGA curves of PD-TPA and M/PD-TPA COFs are shown in Figure S1e–g. PD-TPA COF shows a weightlessness rate of 5.93% before 400 °C caused by the evaporation of surface moisture and solvent molecules.^44^ After 400 °C, there is a remarkable weight loss, indicating that the PD-TPA COF has a good thermal stability before 400 °C.^41^ With the coordination of metal ions, Ni/PD-TPA COF and Cr/PD-TPA COF are stable up to 387 and 176 °C, respectively (Figure S1f,g), which are slightly lower than the pristine PD-TPA COF. The coordination of metal ions generally leads to a decrease in thermal stability, which is consistent with previous reports.^45,46^

The morphologies of the PD-TPA and M/PD-TPA COFs are observed by SEM and TEM imaging. PD-TPA COF exhibits a flower-like shape composed of loosely packed flakes with small sizes of spheroids (Figure 1a), and the lamellar structure remains unchanged after the coordination of metal ions. Ni/PD-TPA COF shows a loose flaky morphology (Figure 1b), while Cr/PD-TPA COF features more loosely clustered flakes (Figure 1c). The TEM image of the PD-TPA COF shows a layered-sheet arrangement (Figure 1d) and is in agreement with the SEM result. Furthermore, in contrast to the PD-TPA COF, the nanoplatelets in Ni/PD-TPA COF are undestroyed, and the staggered layer distance between nanoplatelets appears to be larger (Figure 1e,f). Notably, obvious lattice fringes are observed in the Cr/PD-TPA COF, suggesting a good crystallinity for clear lattice fringes (Figure 1h,i). The elemental mapping analysis (EDS) vividly demonstrates a more uniform dispersion of Ni in the Ni/PD-TPA COF (Figure 1g) compared to the Cr distribution in the Cr/PD-TPA COF (Figure 1j). Moreover, PXRD analysis of PD-TPA COF exhibits three diffraction peaks at 7.8°, 10.2°, and 20.7°, which can be assigned to (200), (210), and (001) facets (Figure 1k).^47^ The strongest (001) facet results from the 2D π–π stacking between adjacent aromatic rings within the COF.^48^ The Ni/PD-TPA COF retains peaks at 8.3° and 20.5°, and the Cr/PD-TPA COF exhibits additional peaks at 12.4°, 14.4°, 28.1°, 32.6°, 34.2°, 35.9°, and 41.0°, suggesting a slight crystallinity change from the coordination of Cr^3+^ that controls the microstructure and may affect the catalytic activity.

**Figure 1:**
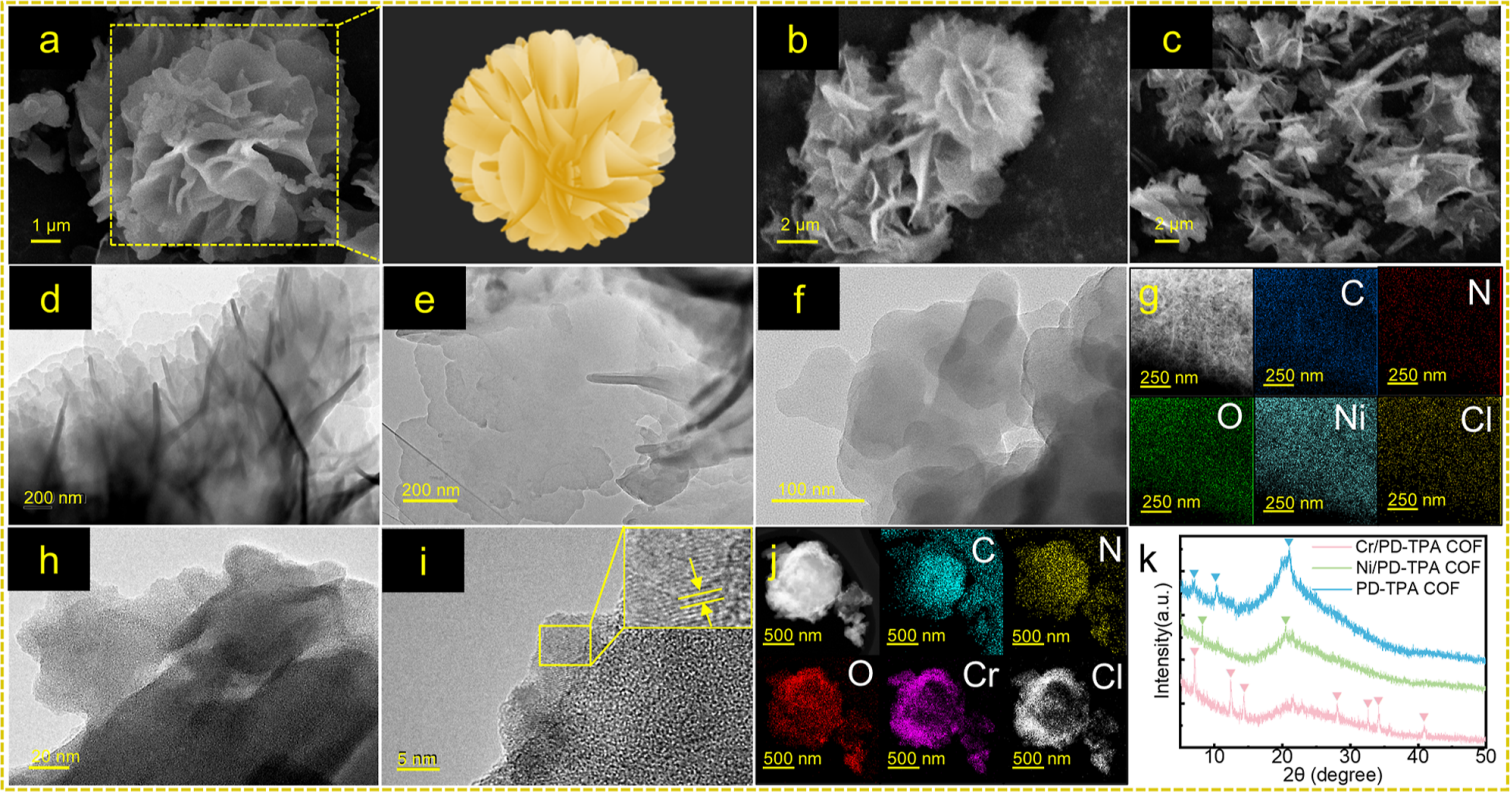
SEM images of (a) PD-TPA COF, (b) Ni/PD-TPA COF, and (c) Cr/PD-TPA COF; TEM images of (d) PD-TPA COF, (e,f) Ni/PD-TPA COF, and (h,i) Cr/PD-TPA COF; elemental mapping of (g) Ni/PD-TPA COF and (j) Cr/PD-TPA COF; (k) XRD profiles of PD-TPA COF and M/PD-TPA COFs.

N_2_ adsorption–desorption isotherms of the PD-TPA and M/PD-TPA COFs are shown in Figure 2. All COFs exhibit type IV isotherms with an H3-type hysteresis loop, suggesting mesoporous structures. The Brunauer–Emmett–Teller (BET) specific surface areas of PD-TPA COF, Ni/PD-TPA COF, and Cr/PD-TPA COF are determined to be 44.5, 35.9, and 7.9 m^2^/g, respectively, and the decrease in M/PD-TPA COFs can be assigned to the coordination of metal ions that partly occupy the mesoporous channels.^49^ According to the pore size distribution (Figure 2b), the PD-TPA and Ni/PD-TPA COF have a single distribution centered around 43.3 and 31.4 nm, respectively. However, the pore size distributions of Cr/PD-TPA COF are not uniform, indicating the presence of a small fraction of microspores centered around 3.7 nm and relatively more abundant mesopores around 36.2 nm.

**Figure 2:**
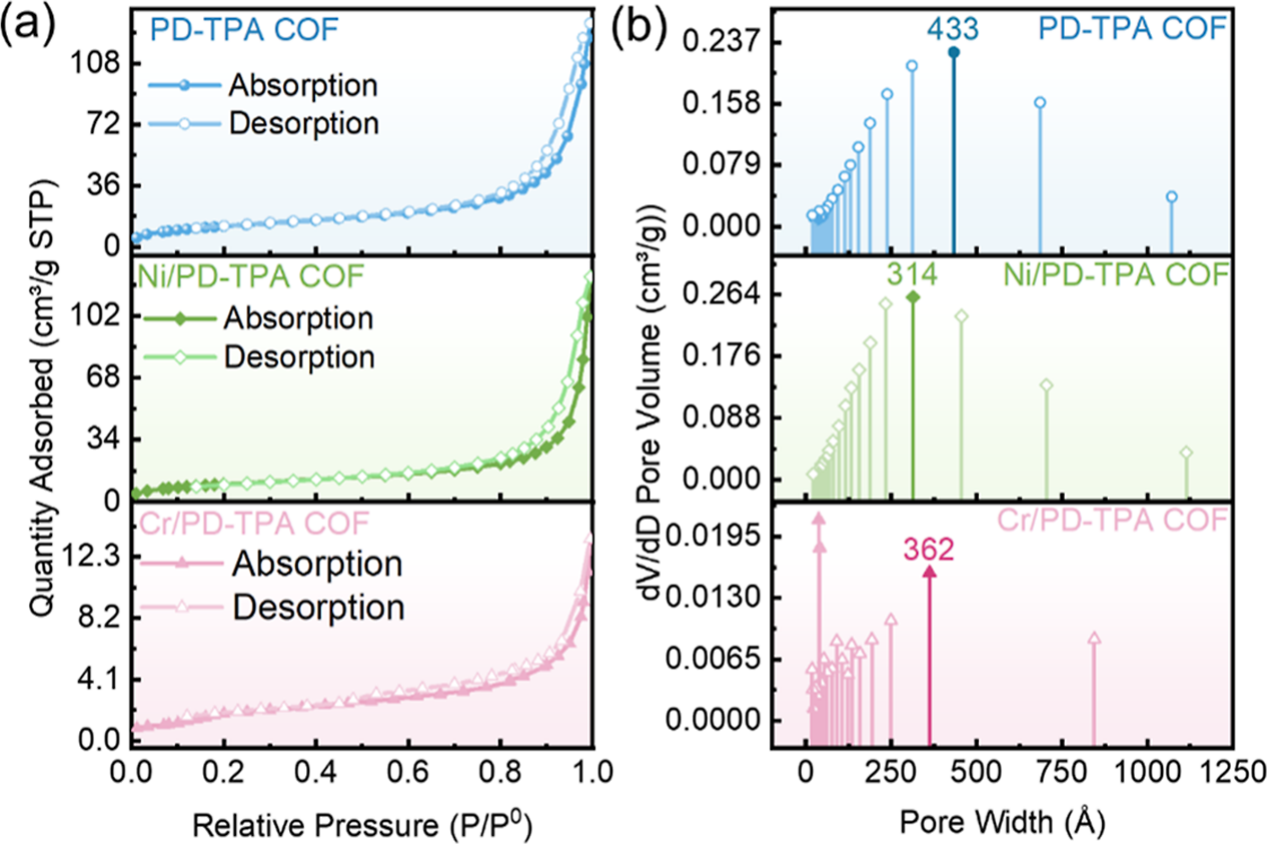
(a) Nitrogen adsorption–desorption isotherms; (b) pore size distribution of PD-TPA and M/PD-TPA COFs.

### Catalytic Performance

The imine-type ligand is one of the most versatile functional groups in coordination chemistry. To enhance the catalytic performance of PD-TPA COF toward ethylene oligomerization, Ni/PD-TPA and Cr/PD-TPA COFs are constructed postmodification. Since there are multiple variables in reaction conditions (e.g., cocatalyst type and dose, reaction temperature, time, and pressure), a high experimental combination of these parameters is initially conducted. A cocatalyst plays an important role in activating the metal center and promoting the coordination of ethylene molecules with the main catalyst to form active intermediates. Three types of alkyl aluminum cocatalysts, including MAO, tri-isobutylaluminum (TIBA), triethylaluminum (TEAL) (Figure 3a), are first employed in ethylene oligomerization. The highest activity of 5.46 × 10^5^ g/(mol Cr·h) and selectivity toward hexene (21.9%) are achieved with TIBA as the cocatalyst when Cr/PD-TPA COF is selected as the catalyst (Figure 3b). In contrast, low activity is observed using MAO, which may be associated with the insufficient reduction capacity of MAO to the Cr/PD-TPA COF in cyclohexane, resulting in its inability to form active species. The highest activity of 3.88 × 10^5^ g/(mol Ni·h) and selectivity toward butylene (86.8%) are achieved with TEAL as the cocatalyst when Ni/PD-TPA COF is used as the catalyst. The dynamic structure of MAO with a typical size exceeding 1 nm, combined with its high cost, imposes significant limitations on its practical applications.^50,51^ In contrast, TEAL, with a smaller van der Waals radius (3.29 Å) compared to TIBA (4.31 Å) and a lower cost, demonstrates greater compatibility with the smaller pore size of Ni/PD-TPA COF. Based on these considerations, TIBA is selected as the preferred cocatalyst for ethylene oligomerization with Cr/PD-TPA COF, while TEAL is more suitable for Ni/PD-TPA COF.

**Figure 3:**
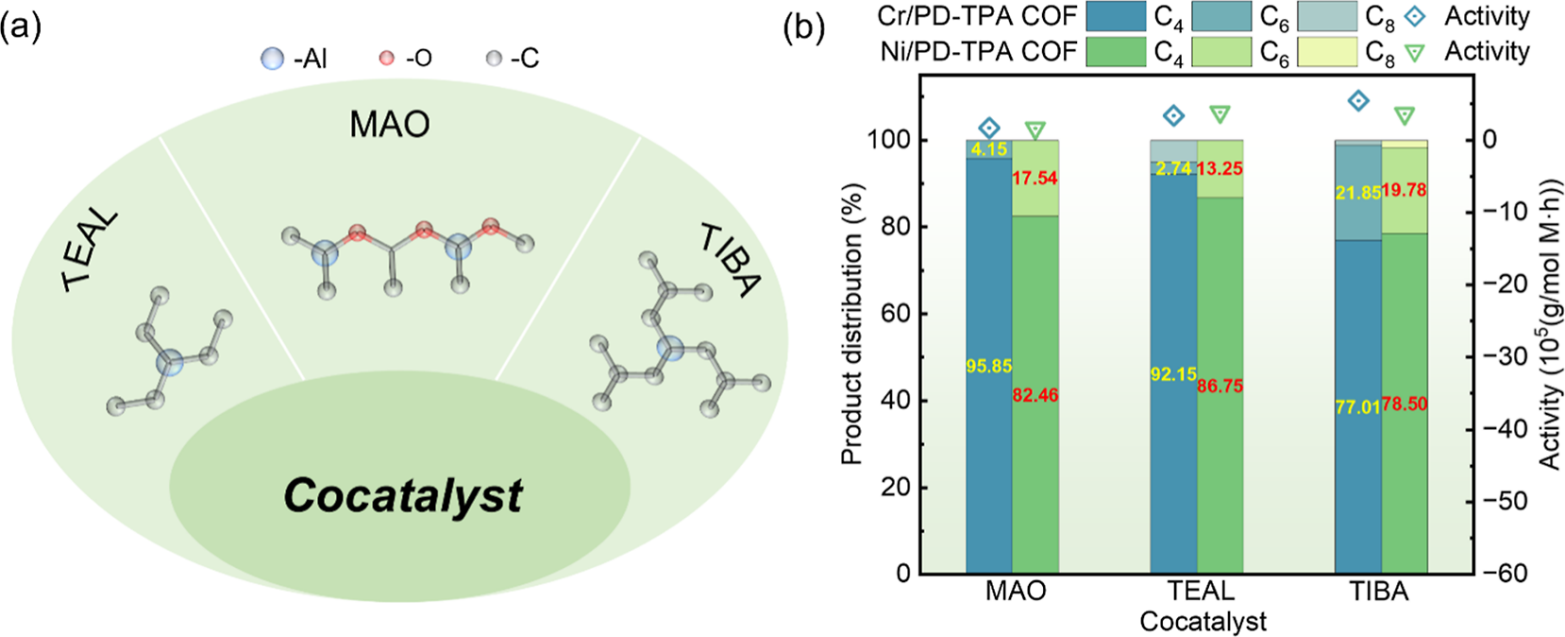
(a) Structure of the cocatalysts; (b) effects of cocatalysts on the catalytic performance.

The effect of the cocatalyst dose is evaluated with various Al/M molar ratios. For Cr/PD-TPA COF, the activity of ethylene oligomerization increases from 2.22 × 10^5^ to 11.00 × 10^5^ g/(mol Cr·h) as the Al/Cr molar ratio increases from 300 to 900 (Figure 4a). Ni/PD-TPA COF exhibits a similar trend in relation to the molar ratio of Al/Ni (Figure 4b). This can be explained by the fact that a higher cocatalyst dose leads to more activated sites for catalysis.^52^ Furthermore, the distribution of the products is also influenced by the Al/M molar ratio. It is found that the selectivity of C_4_ olefin initially increases and then decreases for Cr/PD-TPA COF, which can be associated with the relative barriers between chain growth and β-H elimination. In contrast, Ni/PD-TPA COF shows a good selectivity toward 1-butene which can be explained by a decreased rate of chain growth and enhanced β-hydride elimination at a higher Al/Ni molar ratio. By altering the reaction temperature, both Cr/PD-TPA and Ni/PD-TPA COFs show a decreased activity from 25 to 55 °C (Figure 4c,d), probably due to the retarded transportation of ethylene to active sites as an effect of slumped ethylene concentration in solution.^53,54^ Moreover, since ethylene oligomerization is an exothermic reaction, a low temperature can promote the reaction equilibrium toward the oligomerization side. In terms of product distribution, elevating the temperature leads to more production of C_4_ for Cr/PD-TPA COF since β-hydrogen elimination is much faster than chain propagation at high temperature.^55,56^ For Ni/PD-TPA COF, the selectivity toward 1-octene initially increases but then decreases. The effect of reaction time on ethylene oligomerization is investigated to gain insights into the lifetime of the active catalytic species (Figure 4e,f). For Cr/PD-TPA COF, the catalytic activities of oligomerization decrease with elevating the reaction time from 10 to 90 min, along with a decrease of the selectivity for 1-butene, indicating that the catalyst system is considered to be of the type of rapid initiation and gradual growth. The activity and the selectivity of the [Ni/PD-TPA COF]/TEAL systems show a trend similar to that with [Cr/PD-TPA COF]/TIBA. Figure 4g,h shows the effect of the reaction pressure on the catalytic performance of M/PD-TPA COFs. With regard to Cr/PD-TPA COF, the activity has its growth from 0.3 to 0.5 MPa, whereas a substantial decline as pressure further increased. Eventually, the use of a pressure of 0.5 MPa affords the highest activity with 9.52 × 10^5^ g/(mol Cr·h). We attribute this trend to ethylene adsorption at 0.5 MPa. The Cr/PD-TPA COF, with a low specific surface area (7.9 m^2^/g), has a limited capacity for further ethylene adsorption at higher pressures. Additionally, EDS (Figure 1j) shows a denser Cr­(III) distribution, which leads to the aggregation of active sites and an increased local Cr­(III) concentration. This aggregation reduces the utilization of Cr active centers and inhibits the adsorption of ethylene, resulting in decreased catalytic activity and lower chain growth at higher ethylene concentrations.^57^ For Ni/PD-TPA COF, catalytic activity increases significantly as pressure rises from 0.3 to 1.0 MPa, reaching a maximum of 1.99 × 10^6^ g/(mol Ni·h) at 1.0 MPa. One possible explanation for the trend in activity can be derived from an increase in the solubility of ethylene as the pressure increased, resulting in the accessibility of more ethylene to the active site.^58^ The resulting product featured a narrow distribution, mainly fixed on C_4_ olefin. The selectivity toward C_4_ olefin gradually increased along with the increase of pressure, considering that the enhanced chain termination rate was one of the possible reasons.

**Figure 4:**
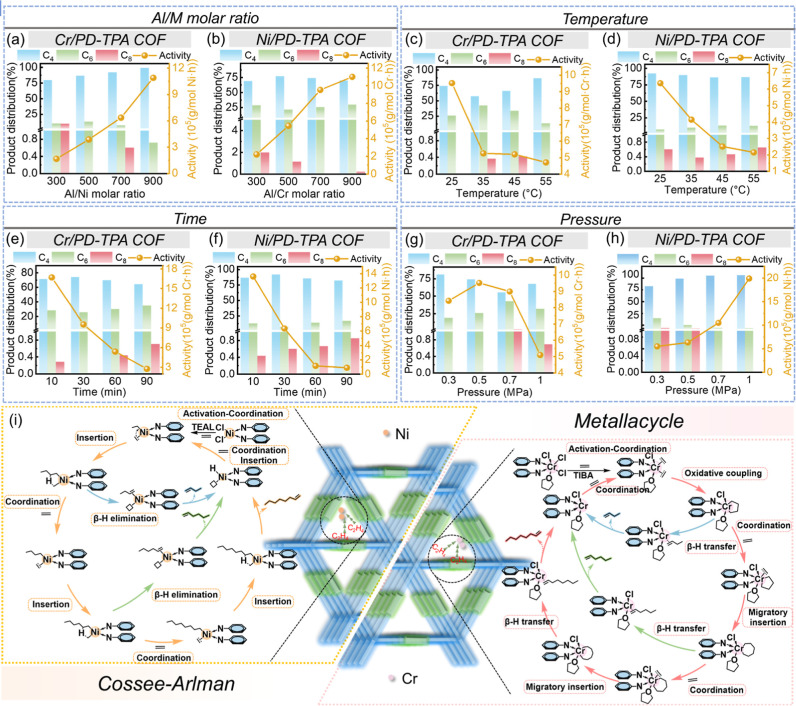
Effects of (a,b) Al/M ratio, (c,d) temperature, (e,f) time, (g,h) pressure on the catalytic performance catalyzed by M/PD-TPA COFs (M = Ni, Cr); (i) proposed mechanism of ethylene oligomerization catalyzed by M/PD-TPA COFs (M = Ni, Cr).

Accordingly, under the optimal conditions (Al/Cr ratio = 700, 25 °C, 10 min, and 0.5 MPa), the highest activity of Cr/PD-TPA COF is 16.70 × 10^5^ g/(mol Cr·h) and the main products are butene (main product) and hexene. The highest catalytic activity of Ni/PD-TPA COF for ethylene oligomerization is 19.90 × 10^5^ g/(mol Ni·h), and the content of C_4_ is up to 98.5% (Al/Ni ratio = 700, 25 °C, 30 min, and 1.0 MPa). Despite the higher activity of the nickel catalyst than that of the chromium catalyst, the latter shows a much higher C_6_ selectivity. Ni/PD-TPA COF exhibits a larger specific surface area and pore diameter than those of Cr/PD-TPA COF, which may facilitate sufficient contact of ethylene with the active centers, resulting in higher activity. Late transition metals, such as Ni­(II), possess higher nuclear charge numbers compared to early transition metals like Cr­(III). This characteristic enables late transition-metal sites to form stronger interactions with β-H, thereby promoting β-H elimination and favoring the production of oligomers.^59^

The Cossee–Arlman mechanism is employed to understand the reaction pathway of ethylene oligomerization over Ni/PD-TPA COF.^60,61^ As shown in Figure 4i, Ni–Cl species are first activated by the cocatalyst TEAL for the formation of Ni-ethyl species with empty orbits. An ethylene molecule coordinates with the Ni­(II) site in the Ni-ethyl species and then inserts into the Ni–C­(ethyl) bond to produce Ni-butyl species. Subsequently, there are two competitive pathways via either the chain propagation by ethylene coordination-insertion to generate higher olefins or β-hydrogen elimination to generate 1-butene. Undoubtedly, leaning toward β-H elimination due to the lack of a large steric hindrance group around the Ni­(II) sites rather than chain propagation facilitated high selectivity of 1-butene in ethylene selective oligomerization catalyzed by Ni/PD-TPA COF. In addition, ethylene dimerization is favored over trimerization and isomerization due to the high energy barrier associated with chain walking, making 1-butene the predominant product.^62^ This finding is consistent with results reported for widely studied MOF-based nickel catalysts in ethylene oligomerization.^14,15,63^ Cr-based catalysts are considered to be more effective in ethylene trimerization via moderate catalytic sites.^6^ A metalacyclic mechanism is used to understand the ethylene oligomerization over Cr/PD-TPA COF.^64^ First, Cr–Cl species are activated by the cocatalyst TIBA for the formation of the Cr center with empty orbits. Afterward, the oxidative coupling of two ethylene monomers over chromium sites yields a chromacyclopentane, which then undergoes reductive elimination via β-hydrogen transfer to produce 1-butene. Another competing pathway follows a ring expansion of chromacyclopentane via coordination and insertion of additional ethylene to form chromacycloheptane, while decomposition of the metallacycle produces 1-hexene. The selectivity of α-olefin is determined by the rate of the reduction/elimination steps compared with that of ring expansion.^65^ The subsequent insertion of ethylene, followed by β-hydrogen transfer, resulted in the formation of 1-octene. However, the expansion of the seven-membered chromacycloheptane ring to form a chromacyclononane is an energetically unfavorable process.^6^ In this study, the confined porous structure of Cr/PD-TPA COF accelerates β-hydrogen transfer relative to ethylene insertion, leading to high selectivity for 1-butene and partial formation of hexene during ethylene oligomerization.^66^

### BBD Evaluation

The single-factor experiment is first employed to identify the main factors in ethylene oligomerization and provide a reasonable numerical range for BBD factors. Since the syntheses of 1-hexene and 1-octene from ethylene are more important in the petroleum industry, this work mainly explores the synergistic effects of cocatalyst dosage, reaction temperature, and time on the activity and the total selectivity toward C_6_ and C_8_ products. Table S3 shows the selected experimental results for the three variables and responses (activity and selectivity). The resulting regression equation is demonstrated by 3D response surface plot to describe the interactions between any two parameters on the activity (*Y*_1_) and the total selectivity toward C_6_ and C_8_ (*Y*_2_).^67^ 3D response surface plots depict an infinite number of permutations involving two test parameters, while another parameter is held at its center point level.

According to multiple regression analysis on the experimental data, the relevance between the variables and activity (*Y*_1_) catalyzed by Cr/PD-TPA and Ni/PD-TPA COFs is illustrated by eqs S2 and S3 (in Supporting Information), respectively. Moreover, the values of the correlation coefficient (*R*) of eqs S1 and S2 are found to be 0.947 and 0.922, indicating a good fit between the model to the experimental data.

The effect of the Al/Cr molar ratio and the reaction temperature on the catalytic activity (*Y*_1_) for Cr/PD-TPA COF is first studied. The Al/Cr molar ratio has a significant and positive effect on the catalytic activity (*Y*_1_), and the catalytic activity increases with a higher Al/Cr molar ratio (Figure 5a). When the Al/Cr ratio increases to 800, the activity reaches a value of close to 7.46 × 10^5^ g/(mol Cr·h). However, the reaction temperature has a negative effect on the catalytic activity (*Y*_1_), and the catalytic activity decreases with further elevated temperatures over 28.2 °C, indicating that the synergistic effect of high Al/Cr and low temperatures can promote ethylene oligomerization. The catalytic activity is influenced by both the reaction time and temperature (Figure 5b), and a single negative effect of time on the catalytic activity is observed. It is noteworthy that high Al/Cr ratios and short reaction times synergistically increase the catalytic activity (Figure 5c). A 3D response surface plot is further plotted as a function of the Al/Ni molar ratio and temperature for Ni/PD-TPA COF (Figure 5d), which indicates that increased temperature can first improve and then decrease activity, presumably because a higher temperature leads to a higher reaction rate with enhanced solubility of ethylene in the solvent but also simultaneously accelerates deactivation of active species. Moreover, with the increase of Al/Ni, the activity poses a negative feedback trend. Similarly, Figure 5f suggests that the highest activity of ethylene oligomerization (7.30 × 10^5^ g/(mol Ni·h)) is obtained under the condition of short reaction time and low Al/Ni molar ratio, which is highly in agreement with the observations in Figure 4b,f.

**Figure 5:**
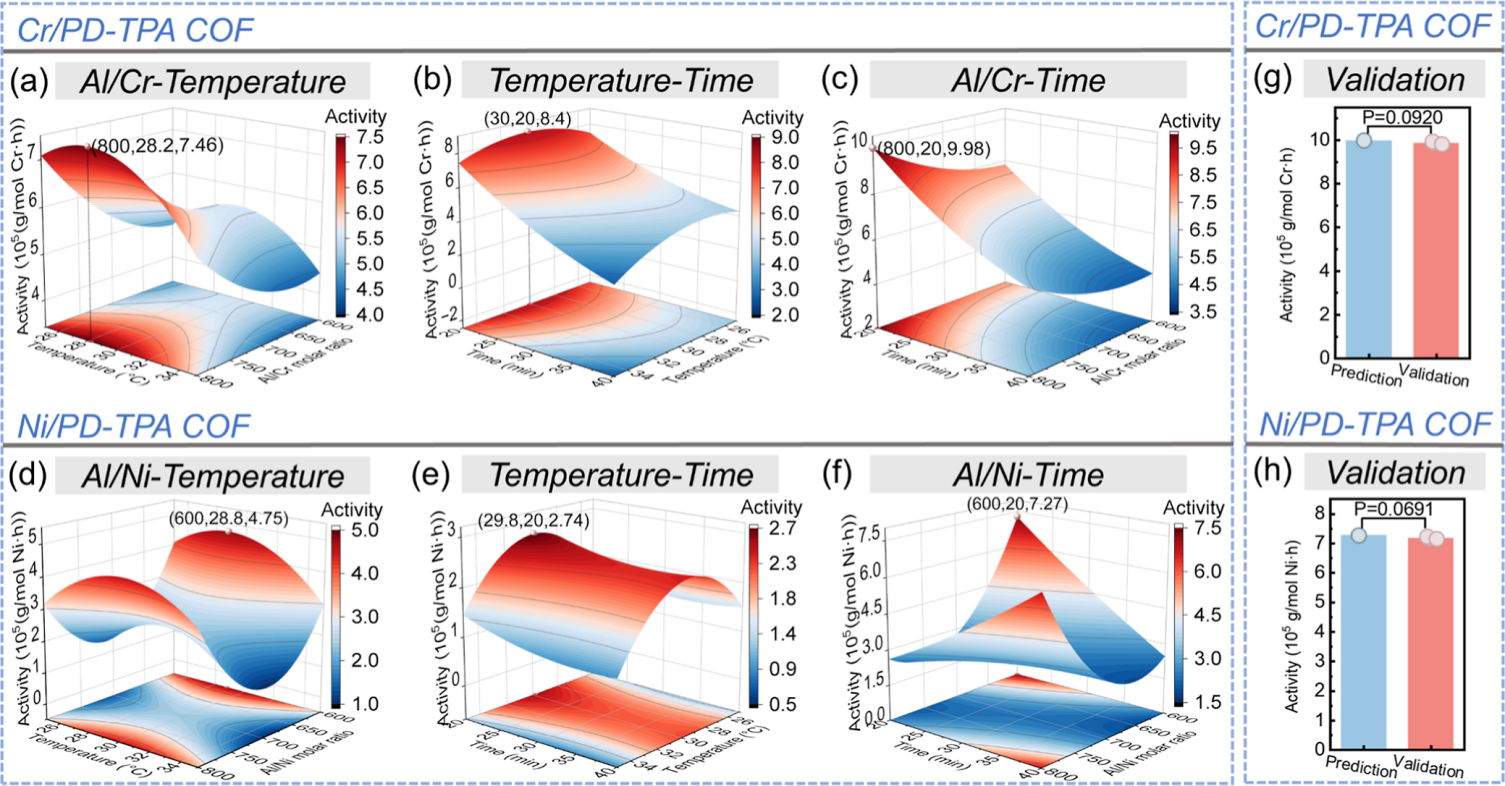
Response surface plot showing the interaction of (a,d) Al/M molar ratio-temperature, (b,e) temperature-time, and (c,f) Al/M molar ratio-time on the activity for ethylene oligomerization; validation experiment of Cr/PD-TPA COF (g) and Ni/PD-TPA COF (h) based on catalytic activity under the optional conditions of BBD optimization.

To evaluate the relevance between the variables and selectivity (*Y*_2_) of Cr/PD-TPA and Ni/PD-TPA COFs, the models of second-order polynomial equations are illustrated by eqs S4 and S5. The correlation coefficient (*R*) of eqs S3 and S4 (in Supporting Information) is calculated as 0.949 and 0.927, respectively, indicating that the model possesses a significant correlation. Figure 6a–c shows the impact of three variables (Al/Cr molar ratio, temperature, time) on the total selectivity toward C_6_ and C_8_. Upon closer observation of Figure 6a, it is found that the increased Al/Cr molar ratio leads to a gradual increase in the total selectivity toward C_6_ and C_8_ for Cr/PD-TPA COF. This trend can be attributed to the enhanced rate of chain growth caused by an increase in the amount of the cocatalyst, which is in agreement with the result in Figure 4a. The maximum total selectivity toward C_6_ and C_8_ is 48.3% at an Al/Cr ratio of 800 and 25 °C, implying that the high Al/Cr and low temperature can favor the selective production of C_6_ and C_8_. Furthermore, the Al/Cr molar ratio is similarly in a single dominant position in the interaction of Al/Cr-temperature on the total selectivity toward C_6_ and C_8_. It is found that a prolonged reaction time and higher Al/Cr ratio can promote the formation of C_6_ and C_8_ (Figure 6c). Figure 6b shows the impact of reaction temperature and time on the total selectivity toward C_6_ and C_8_, and the fact that more reaction time is conducive to the formation of C_6_ and C_8_ has been again verified. Figure 6d–f shows the impacts of three variables (Al/Ni molar ratio, temperature, time) on the total selectivity toward C_6_ and C_8_ for Ni/PD-TPA COFs. It is found that the optimal conditions (Al/Ni ratio = 700, 35 °C, 20 min) resulted in a predicted total selectivity of 49.2% toward C_6_ and C_8_. In addition, a longer reaction time and higher reaction temperature can promote the formation of olefins with higher carbon numbers, resulting in wide product distribution and increased separation difficulty, which is consistent with the results of the single-factor effect on the catalytic performance. Based on the above results, it is found that the BBD as a statistical tool can quickly establish optimal process parameters through an accurate model. The number of BBD runs is less than that of single-factor experiments, which achieves economic practicality by minimizing the operating costs and labor consumption.

**Figure 6:**
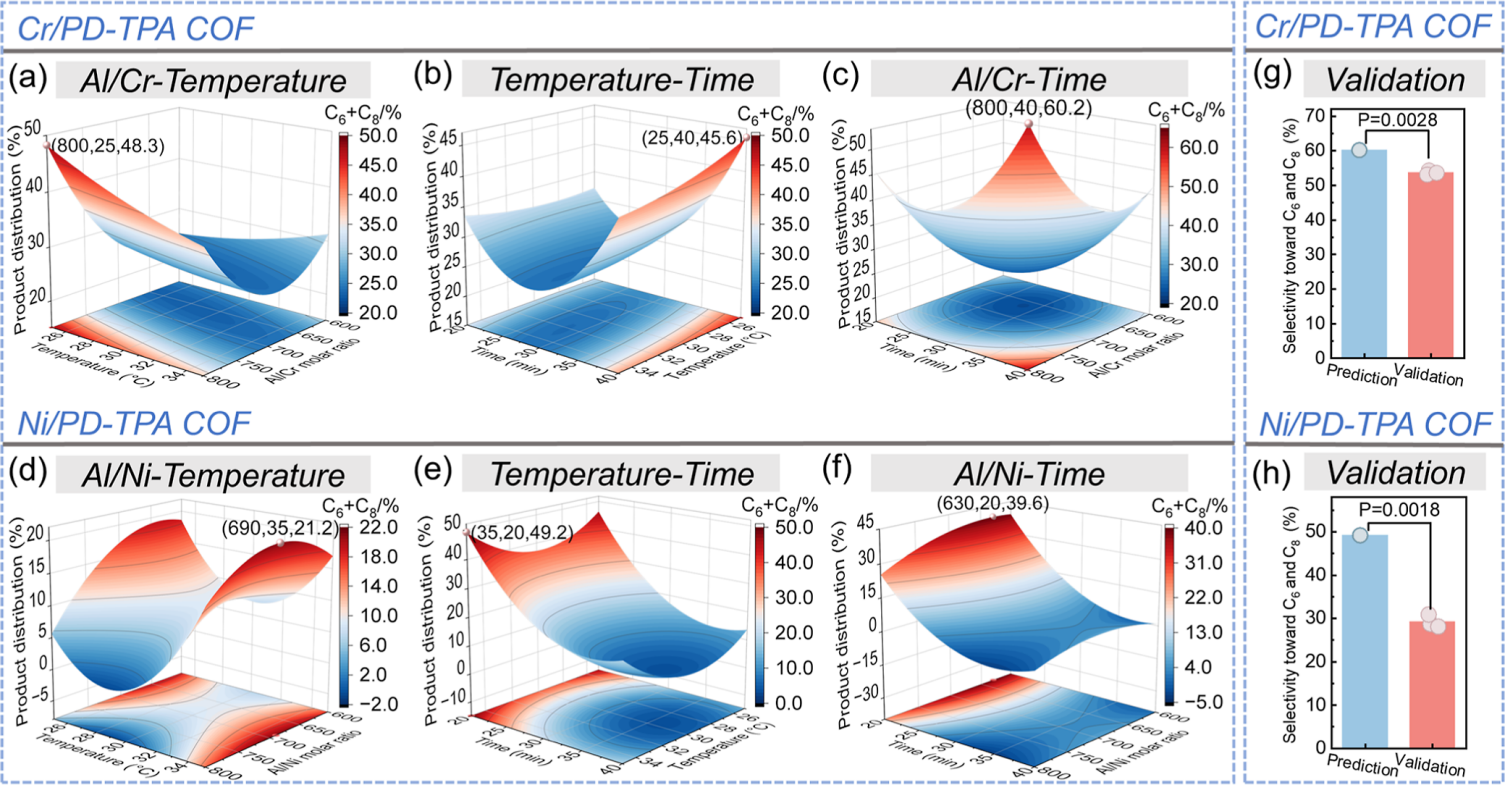
Response surface plots showing the interaction of (a,d) Al/M molar ratio-temperature, (b,e) temperature-time, and (c,f) Al/M molar ratio-time on the total selectivity toward C_6_ and C_8_ for ethylene oligomerization; validation experiment of Cr/PD-TPA COF (g) and Ni/PD-TPA COF (h) based on the total selectivity toward C_6_ and C_8_ under the optional conditions of BBD optimization.

To verify the accuracy of the regression model, three repeated experiments are conducted under the optimal conditions predicted by the regression template, as shown in Figures 5g,h and 6g,h. As shown in Figure 5g,h, the results for verification of catalytic activity revealed that the activities of the three experimental groups closely aligned with the predicted values. The calculated “significant difference *P*-value” is 0.0920 for Cr/PD-TPA COF, and 0.0691 for Ni/PD-TPA COF, indicating no statistically significant difference between the predicted and experimental values (*P* > 0.05). It shall be specifically noted that significant difference is an evaluation of the difference in data in statistics, and *P* > 0.05 indicates that the difference is not significant usually.^68^ Regarding product selectivity, the verification results exhibited some deviation from the predicted values. However, it is observed that the average selectivity for Cr/PD-TPA COF is approximately 53.67%, while that for Ni/PD-TPA COF is around 29.24%, consistent with the selectivity trends obtained from the single-factor optimization process. These results confirm that even under optimized conditions, the inherent nature of the active centers determines that Cr/PD-TPA COF favors ethylene trimerization, whereas Ni/PD-TPA COF predominantly facilitates ethylene dimerization. Despite this deviation, the optimization of product selectivity through the BBD model highlights the multifactorial influences on selectivity outcomes. This finding aids in establishing optimal process conditions and underscores the high applicability of the regression model for M/PD-TPA COFs in ethylene oligomerization.

### Recycle Experiment

Traditional homogeneous catalysts for ethylene oligomerization are easily deactivated, which raises concerns about the sustainable conversion of ethylene to α-olefins. Heterogeneous catalysis provides an alternative strategy for sustainable and low-cost production of valuable chemicals, where the recycling of catalysts is a crucial step. Herein, the performance of M/PD-TPA COFs after recycling is examined to confirm the reusability of the catalysts. The materials remain active up to three catalytic cycles, providing advantages comparable to and even higher than previous reports (Figure 7b).^30,64^ The variation of product selectivity for Ni/PD-TPA COF is similar to that for Cr/PD-TPA COF in the recycling. With an increase in recycle numbers, the selectivity of Ni/PD-TPA COF toward hexene decreases. This might be attributed to the blocking of residual oligomers adsorbed on the surface of the catalyst after each cycle and a reduction in the accessibility of active nickel species to ethylene, which may inhibit the formation of high-carbon α-olefins and increased the selectivity of low-carbon α-olefins.^30^ In terms of Cr/PD-TPA COF, the selectivity toward hexene is enhanced with increased recycle numbers. It is noteworthy that the experimental system produced trace amounts of polymers after the third cycle. The cocatalyst (TIBA) is responsible for this phenomenon since it can trigger chain transfer reactions and generate additional polymeric active sites for the formation of long-chain polymers.

**Figure 7:**
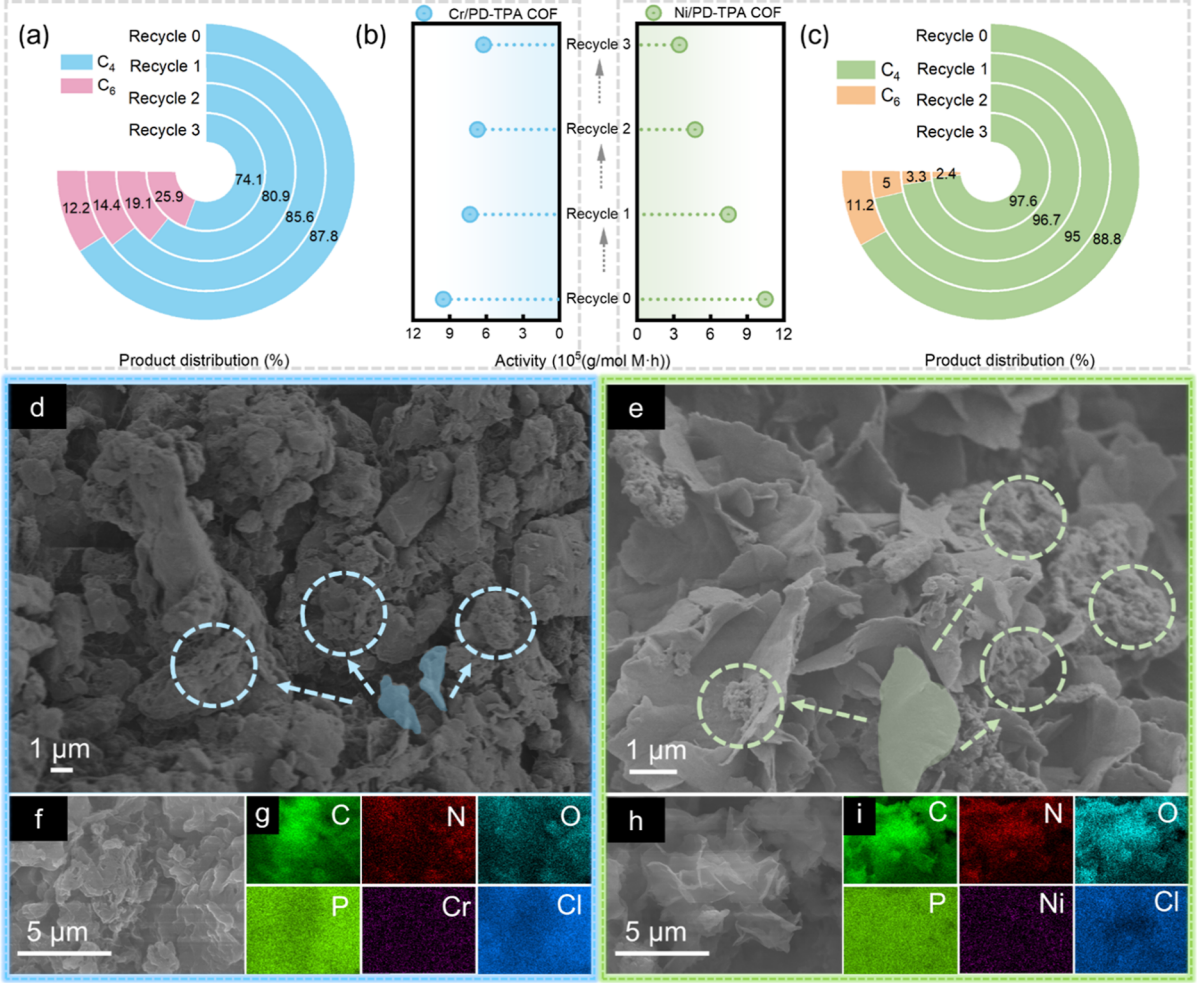
Product distribution of (a) Cr/PD-TPA COF and (c) Ni/PD-TPA COF in the recycle experiment; (b) activity of M/PD-TPA COFs in the recycle experiment; SEM images of (d) Cr/PD-TPA COF and (e) Ni/PD-TPA COF after the third cycle; HAADF-STEM image of (f) Cr/PD-TPA COF and (h) Ni/PD-TPA COF, corresponding EDS mapping images of (g) Cr/PD-TPA COF and (i) Ni/PD-TPA COF.

The recovered M/PD-TPA COFs are further characterized by FTIR and SEM to confirm the differences in functional groups and morphologies. The FTIR analysis shows that the related functional groups are almost preserved after recycling (Figure S2). For SEM, the morphological transformation from flake to block agglomeration is found in both catalysts. With increasing cycle times, catalyst agglomeration occurs gradually, which is attributed to the residual oligomers adsorbed on the surface of the catalyst after the reaction and may restrict the access of ethylene to active sites. Furthermore, the chromium catalyst exhibits a high degree of agglomeration, which may be a result of the polymer coating on the catalyst, indicating its contribution to the increased level of hexene. The uniform dispersion of metal active centers is analyzed through EDS to determine the metal distribution throughout the surface of the M/PD-TPA COFs. The EDS images (Figure 7f–h) demonstrate that Cr­(III) and Ni­(II) are homogeneously distributed over the surface of the materials.

A comparison for catalytic performances and reaction conditions of this work and previous publications is shown in Table S4, including COFs,^31^ MOFs,^69^ magnetic nanoparticles,^70^ mesoporous silica,^71^ and carbon nanotubes,^72^ as shown in Table S4. It is found that M/PD-TPA COFs in this work exhibit comparable or even higher ethylene oligomerization activity (Cr/PD-TPA COF: 2.22 × 10^5^ g/(mol Cr·h), Ni/PD-TPA COF: 1.68 × 10^5^ g/(mol Ni·h)) at ambient temperature, low pressure, and significantly lower cocatalyst additions compared with above supported catalysts under optimal conditions. The high activity and mild conditions demonstrate the promise of M/PD-TPA COFs as an efficient platform for ethylene oligomerization with higher atomic efficiency and diminished waste.

## Conclusions

In this work, an imine-linked COF material (PD-TPA COF) is synthesized from (PD-(NH_2_)_6_) and TPA, and two metal species are loaded into a well-defined pore structure and coordinated with C=N groups. The resulting Ni/PD-TPA and Cr/PD-TPA COFs are shown to be highly effective in catalytic ethylene oligomerization. Single-factor experiments demonstrate that the Cr/PD-TPA COF has the highest activity of 16.70 × 10^5^ g/(mol Cr·h) and the highest selectivity of butene and hexane upon activation with TIBA, and the Ni/PD-TPA COF has the highest catalytic activity of 19.90 × 10^5^ g/(mol Ni·h) and the highest selectivity of butane with TEAL as the cocatalyst. The confinement effect and the metal active centers cooperatively modulate the performance of ethylene oligomerization, and the large specific surface and pore sizes are beneficial for the effective contact of olefins with the active sites. Further efforts are made in endeavoring to optimize the multiple interactions of experimental parameters (dosage of cocatalyst, temperature, and time) on the catalysis performance, and statistical models are established for process parameters and performance via BBD tools. Moreover, a high correlation coefficient (*R* > 0.9) verifies the reliability of the model and the synergistic effect of reaction parameters in ethylene oligomerization. In addition, M/PD-TPA COFs can be easily recovered and reused for several cycles, retaining significant catalytic activities. Finally, given that the catalysis occurs at ambient temperature and low pressure, along with the minimal quantity of cocatalyst, the high activity shown by M/PD-TPA COFs compared with the catalyst with other supports enables significant reduction in materials usage and waste. In summary, the catalysts boast environmental acceptability, economic viability, and recyclability.

## Supplementary Material



## Notes

Experimental section (materials and characterization methods, ethylene oligomerization tests, recycle experiment, and BBD processing); variables and their levels in the BBD, Box–Behnken experimental design, experimental runs and observed responses for the BBD, and comparisons of the current systems to the literature catalysts; characterization spectra including FTIR, ^13^C SSNMR, XPS, and TGA of both PD-TPA COF and M/PD-TPA COFs, as well as FTIR spectra of the spent catalysts after the third catalytic cycle; and all regression equation derived from the Box–Behnken response surface methodology (PDF)
