# Supplementary material for: Catalytic Ethylene Oligomerization over Imine-Linked Covalent-Organic Frameworks with Coordinative Ni(II) and Cr(III)
Source: ACS Sustain Chem Eng. 2025 May 9;13(20):7520–31. doi: 10.1021/acssuschemeng.5c01284 (PMC12117602; doi:10.1021/acssuschemeng.5c01284)
Supplement: Supplementary file 1 [file sc5c01284_si_001.pdf]

Supporting Information

Catalytic ethylene oligomerization over imine-linked covalent-organic frameworks with coordinative Ni(II) and Cr(III)

Lijun Guo,[a] Yingchuan Zhang,[b]* Wei Chen,[a] Yang Li,[a] Da Song,[c] Weiwei Yang,[a] Jin Huang,[a] Feng Li,[a] Cuiqin Li [a]* and Zhengxiao Guo [b]*

[a] Provincial Key Laboratory of Polyolefin New Materials, College of Chemistry & Chemical Engineering, Northeast Petroleum University, Daqing 163318, PR China

[b] Department of Chemistry, The University of Hong Kong, Hong Kong SAR, PR China

[c] Guangzhou Institute of Energy Conversion, Chinese Academy of Sciences, Guangzhou 510640, PR China

Corresponding authors: zxguo@hku.hk; yczhangh@connect.hku.hk; dqpilicuiqin@126.com

The number of pages, tables, and equations included in the supporting information file:

Number of pages: 14 Number of tables: 4

Number of equations: 5 Number of figure: 2

Table of contents

Experimental section **S3**

Materials and characterizations.......... S3

Oligomerization reaction S4

Recycle experiment S5

BBD processing S5

Supplementary tables **S7**

Table S1 Variables and their levels in the Box-Behnken design.......... S7

Table S2 Box-Behnken experimental design S7

Table S3 Experimental runs and observed responses for the Box-Behnken design S8

Table S4 Comparisons of the current systems to the literature catalysts S9

Supplementary Figures.......... **S10**

Figure S1. (a) FTIR spectra of PD-TPA COF and M/PD-TPA COFs (M = Ni, Cr); (b) ^{13}C SSNMR spectrum of PD-TPA COF; (c) and (d) XPS spectra of Ni/PD-TPA COF and Cr/PD-TPA COF; (e) (f) and (g) TGA/DTG curves of PD-TPA COF, Ni/PD-TPA COF and Cr/PD-TPA COF. S10

Figure S2. FTIR of the spent catalysts after the 3rd cycles S11

Supplementary equations.......... **S12**

Reference.......... **S13**

Experimental section

Materials and characterizations

Methylaluminoxane (MAO, 10 wt% in toluene), triisobutylaluminum (TIBA, 1 M in hexane), triethylaluminium (TEAL, 1 M in hexane) were purchased from Sigma-Aldrich. Terephthalaldehyde and chromium(III) chloride tetrahydrofuran complex ($CrCl_3(THF)_3$) were provided by Shanghai Aladdin Biochemical Technology Co., Ltd. All solvents including ethanol, ethanol, dichloromethane, tetrahydrofuran, toluene and 1,4-dioxane were purchased from Tianjin kemio Chemical Reagent Co., Ltd. PD-$(NH_2)_6$ [1] and (DME)$NiCl_2$ were synthesized according to the previous literature.[2]

Infrared (IR) spectra were recorded on Nicolet FTIR 750 infrared spectrometer using the KBr pellets method. ^{13}C solid-state nuclear magnetic resonance spectrum (^{13}C SSNMR) was collected at ambient pressure on Bruker AV-400 NMR spectrometer (Bruker, Switzerland). X-ray photoelectron spectroscopy (XPS) measurements were carried out on Thermo Fisher Scientific ES-CALAB 250Xi X-ray photoelectron spectrometer with an Al Ka X-ray source (1486.6 eV photons) at an operating voltage of 20 kV and a current of 10 mA. Powder X-ray diffraction (PXRD) patterns were obtained from D/MAX-2200 diffractometer using Cu–Kα radiation. Field-emission scanning electron microscopy (FE-SEM) images were collected on Zeiss SIGMA operated at an acceleration voltage of 10 kV. Transmission electron microscopy (TEM) was performed using JEOL JEM-2100F. EDS energy spectrometer manufactured by American Thermoelectric Noran.

Thermal gravimetric analysis (TGA) was carried out on SDT Q600 from 30 to 900 °C under nitrogen atmosphere at a heating rate of 10 °C/min. Nitrogen adsorption-desorption isotherms were obtained using a Micrometrics ASAP 2460 surface area and porosity analyzer at 77 K. Before that, the samples were degassed in vacuum at 120 °C for more than 10 h. The metal content of the synthesized Ni/PD-TPA COF and Cr/PD-TPA COF was determined using ICPS-7510 spectrometer, where 10 mg Ni/PD-TPA COFand Cr/PD-TPA COF were digested with 2 mL HCl, 6 mL HNO_3 and 5 mL HF, and the resulting solution was diluted with deionized water. GC analysis for evaluating the performance of ethylene oligomerization was conducted on a Fuli GC 9720 equipped with a flame ionization detector (FID) and a 50m (0.2 mm i.d., 0.5μm film thickness) HP-PONA column.

Oligomerization reaction

Ethylene oligomerization was carried out in a 100 mL stainless steel autoclave equipped with a magnetic stir bar. The reactor was first purged with nitrogen three times. When the temperature reached to the desired value, nitrogen was removed and ethylene was introduced. Then, the appropriate amounts of solvent, cocatalyst and precatalyst were injected into the reactor with a syringe sequentially. The mixture was vigorously stirred for the desired time at the appropriate pressure of ethylene. After the reaction, the reaction mixture was cooled in an ice-water bath and the gas was collected with gasbags. The liquid was quenched by 10% HCl in ethanol. In the initial entry, the reaction conditions were: 20 mL cyclohexane, 0.5 μmol catalyst, Al/M ratio of 500, 25 °C,

0.5 MPa, 30 min.

Gas and liquid products were quantified by GC (Fuli GC9720) equipped with HP-PONA capillary column (60 m long, 0.25 mm ID, 0.5 μm film thickness). The analysis conditions were 50 °C for 5 minutes, heated at 10 °C/min until the temperature reached 140 °C, then heated at 5 °C/min until the temperature reached 240 °C and maintained at the temperature for 5 min.

Recycle experiment

The reaction was quenched by the acidified ethanol solution and the solid catalysts were collected by filtration. Then the solid was suspended in cyclohexane by ultrasonic dispersion and stirred for 1 h at 25 ℃. The recycled catalyst was filtrated and washed with ethanol and cyclohexane, respectively. The catalyst was dried under vacuum at 60 ℃ for 4 h and reused in the subsequent recycle experiment of ethylene oligomerization.

BBD processing

BBD is an independent, rotatable quadratic design based on a three-level incomplete factorial design,[3] which are carried out to ascertain the nonlinear relationship between indicators and factors as a statistical tool. A polynomial regression model (**Equation S1**) is employed to quantify the influence of the variables and system response,

$$Y = \beta_0 + \beta_1 x_1 + \beta_2 x_2 + \beta_3 x_3 + \beta_{12} x_1 x_2 + \beta_{13} x_1 x_3 + \beta_{23} x_2 x_3 + \beta_{11} x_1^2 + \beta_{22} x_2^2 + \beta_{33} x_3^2$$

(**Equation S1**)

where Y is the dependent variable, x_1, x_2 and x_3 are the independent variables. Besides, β_0 is a constant variable of the model. The regression coefficients (β_1, β_2, and β_3), (β_{12}, β_{13}, and β_{23}) and (β_{11}, β_{22}, and β_{33}) are linear, interaction, and quadratic coefficients of the model, respectively. In the present study, a three-level, three-factor Box-Behnken experimental design is applied to determine and optimize process parameters affecting ethylene oligomerization and to investigate the interaction between them. Independent variables evaluated in this study were the Al/M molar ratio (M=Cr or Ni, x_1), temperature (x_2) and time (x_3) when the activity of oligomerization reactions (Y_1) and the total selectivity towards C_6 and C_8 (Y_2) in the product were the responses. The detailed experimental range and levels of the independent variables coded as low (–1), medium (0) and high (+1) were given in **Table S1**. Data Processing System (DPS) ware was used to establish the Box-Behnken design and process statistical data analysis [4] and a total of 15 experiments were created and carried out oligomerization reactions at random, and the experimental design matrix was presented in **Table S2**.

Supplementary tables

Table S1 Variables and their levels in the Box-Behnken design

Code	Variables	Levels in Box–Behnken design		
		Low(-1)	Middle(0)	High(+1)
x_1	Al/M	600	700	800
x_2	Temperature (℃)	25	30	35
x_3	Time (min)	20	30	40

Table S2 Box-Behnken experimental design

Test number	Al/M<br>x_1	Temperature<br>x_2	Time<br>x_3
1	600	25	30
2	600	35	30
3	800	25	30
4	800	35	30
5	600	30	20
6	600	30	40
7	800	30	20
8	800	30	40
9	700	25	20
10	700	25	40
11	700	35	20
12	700	35	40
13	700	30	30
14	700	30	30
15	700	30	30

Table S3 Experimental runs and observed responses for the Box-Behnken design

Test NO.	Al/Cr x_1	Temperature x_2	Time x_3	Cr/PD-TPA COF[a]		Ni/PD-TPA COF[b]	
				Y_1(Activity×10^5)	Y_2(C_{6+8}/%)	Y_1(Activity×10^5)	Y_2(C_{6+8}/%)
1	600	25	30	5.65	72.87	3.88	13.19
2	600	35	30	4.23	64.64	2.38	13.98
3	800	25	30	7.30	55.76	3.74	8.55
4	800	35	30	4.86	65.30	3.69	20.67
5	600	30	20	7.88	66.70	8.49	36.22
6	600	30	40	3.48	61.49	2.02	12.12
7	800	30	20	10.7	50.22	3.05	14.11
8	800	30	40	5.58	36.82	5.54	3.06
9	700	25	20	6.51	67.12	0.68	54.44
10	700	25	40	4.12	53.35	2.14	7.82
11	700	35	20	7.60	67.55	0.81	54.97
12	700	35	40	3.56	61.80	2.01	10.56
13	700	30	30	6.18	82.39	1.70	8.90
14	700	30	30	6.39	69.55	3.13	8.55
15	700	30	30	5.14	74.14	2.34	8.40

[a]Condition : 0.5 μmol precatalyst, 20 mL cyclohexane, TIBA as co-catalyst, 30 min.

[b]Condition : 0.5 μmol precatalyst, 20 mL cyclohexane, TEAL as co-catalyst, 30 min.

Table S4 Comparisons of the current systems to the literature catalysts

Catalyst	Al/M	Temperature °C	Time min	Pressure MPa	Activity 10^5 g/mol M·h	Reference
Cr/PD-TPA COF	300	25	30	0.5	2.22	This work
Ni/PD-TPA COF	300	25	30	0.5	1.68	This work
Ni@MABD-COF	700	25	60	0.7	0.76	[5]
Ni@MOF	800	50	30	1.0	1.81	[6]
Ni2@Fe_3O_4	200	120	120	2.0	0.98	[7]
SCn@MWCNTs	700	25	30	0.7	0.83	[8]
SBA-15	200	30	60	1.0	0.35	[9]

Supplementary figures

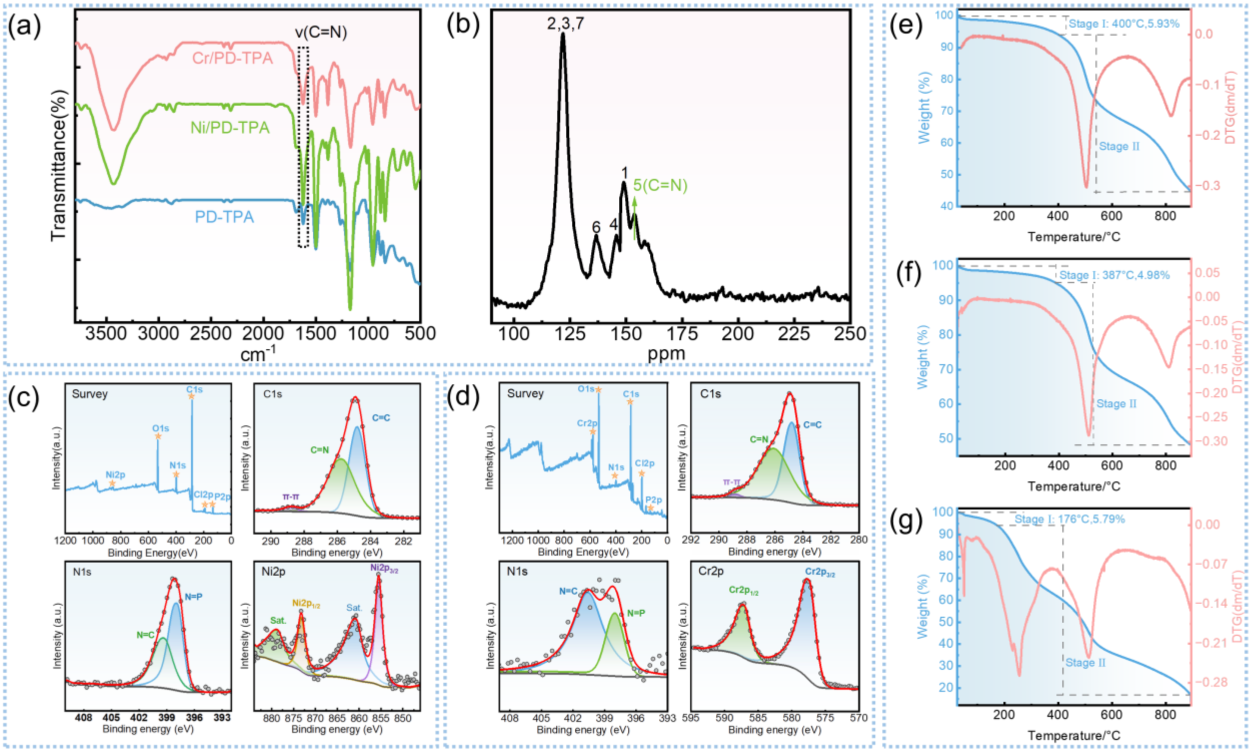

Figure S1. (a) FTIR spectra of PD-TPA COF and M/PD-TPA COFs (M = Ni, Cr), (b) ^{13}C SSNMR spectrum of PD-TPA COF, (c) and (d) XPS spectra of Ni/PD-TPA COF and Cr/PD-TPA COF, (e) (f) and (g) TGA/DTG curves of PD-TPA COF, Ni/PD-TPA COF and Cr/PD-TPA COF.

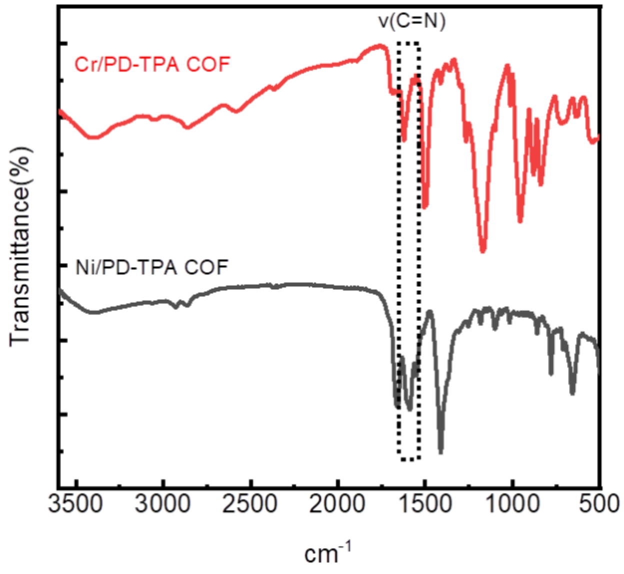

Figure S2. FTIR of the spent catalysts after the 3rd cycles

Supplementary equations

Equation S2

$Y_1(\mathrm{Cr}) = -16.7945833 - 0.0451417\mathrm{x}_1 + 2.7482500\mathrm{x}_2 - 0.1091250\mathrm{x}_3 - 0.0005100\mathrm{x}_1\mathrm{x}_2 -$

$0.0001800\mathrm{x}_1\mathrm{x}_3 - 0.0082500\mathrm{x}_2\mathrm{x}_3 + 0.0000535\mathrm{x}_1^2 - 0.0371167\mathrm{x}_2^2 + 0.0047208\mathrm{x}_3^2$ (R=0.947)

Equation S3

$Y_1(\mathrm{Ni}) = 133.2287500 - 0.3977125\mathrm{x}_1 + 2.2917500\mathrm{x}_2 - 1.6572500\mathrm{x}_3 + 0.0007250\mathrm{x}_1\mathrm{x}_2 -$

$0.0022400\mathrm{x}_1\mathrm{x}_3 - 0.0013000\mathrm{x}_2\mathrm{x}_3 + 0.0002199\mathrm{x}_1^2 - 0.0466500\mathrm{x}_2^2 + 0.0018625\mathrm{x}_3^2$ (R=0.922)

Equation S4

$Y_2(\mathrm{Cr}) = 406.4387500 - 1.0784250\mathrm{x}_1 + 4.6837500\mathrm{x}_2 - 6.8756250\mathrm{x}_3 - 0.0088850\mathrm{x}_1\mathrm{x}_2 +$

$0.0020475\mathrm{x}_1\mathrm{x}_3 - 0.0401000\mathrm{x}_2\mathrm{x}_3 + 0.0009683\mathrm{x}_1^2 + 0.0414000\mathrm{x}_2^2 + +0.1187000\mathrm{x}_3^2$

(R=0.949)

Equation S5

$Y_2(\mathrm{Ni}) = 513.6808300 + 0.4020167\mathrm{x}_1 - 29.1535000\mathrm{x}_2 - 11.8757500\mathrm{x}_3 +$

$0.00566500\mathrm{x}_1\mathrm{x}_2 + 0.0032625\mathrm{x}_1\mathrm{x}_3 + 0.0110500\mathrm{x}_2\mathrm{x}_3 - 0.0005045\mathrm{x}_1^2 + 0.4210167\mathrm{x}_2^2 +$

$0.1280542\mathrm{x}_3^2$ (R=0.927)

Reference

(1) Guo, L.; Huang, J.; Chen, N.; Hu, Y.; Li, M.; Li, F.; Li, C. Synthesis and ethylene oligomerization behavior of trinuclear nickel complex with phosphorus dendrimer. *Transition Met. Chem.* **2022**, *47* (1), 1-9. DOI: 10.1007/s11243-021-00483-3.

(2) Kermagoret, A.; Braunstein, P. Mono- and dinuclear nickel complexes with phosphino-, phosphinito-, and phosphonitopyridine ligands: synthesis, structures, and catalytic oligomerization of ethylene. *Organometallics* **2008**, *27* (1), 88-99. DOI: 10.1021/om7008759.

(3) Ferreira, S. L. C.; Bruns, R. E.; Ferreira, H. S.; Matos, G. D.; David, J. M.; Brandao, G. C.; da Silva, E. G. P.; Portugal, L. A.; dos Reis, P. S.; Souza, A. S.; et al. Box-Behnken design: an alternative for the optimization of analytical methods. *Anal. Chim. Acta* **2007**, *597* (2), 179-186. DOI: 10.1016/j.aca.2007.07.011.

(4) Tang, Q.-Y.; Zhang, C.-X. Data Processing System (DPS) software with experimental design, statistical analysis and data mining developed for use in entomological research. *Insect Sci.* **2013**, *20* (2), 254-260. DOI: 10.1111/j.1744-7917.2012.01519.x.

(5) Li, D.; Guo, L. J.; Li, F.; Huang, J.; Li, J. H.; Li, M.; Li, C. Q. Synthesis and catalytic behavior of nickel heterogenized in covalent organic frameworks as precatalysts in ethylene oligomerization. *Microporous Mesoporous Mater.* **2022**, 338, 13. DOI: 10.1016/j.micromeso.2022.111979.

(6) Chen, L.; Jiang, Y.; Huo, H.; Liu, J.; Li, Y.; Li, C.; Zhang, N.; Wang, J. Metal-organic framework-based composite Ni@MOF as heterogenous catalyst for ethylene trimerization. *Appl. Catal., A* **2020**, 594. DOI: 10.1016/j.apcata.2020.117457.

(7) Ngcobo, M.; Ojwach, S. O. Ethylene oligomerization reactions catalyzed by recyclable Fe(II), Ni(II) and Co(II) complexes immobilized on Fe_3O_4 magnetic nanoparticles. *Mol. Catal.* **2021**, 508. DOI: 10.1016/j.mcat.2021.111583.

(8) Li, Y.; He, C.; Song, D.; Fan, M.; Guo, L.; Zhai, X.; Li, F.; Li, C.; Huang, Z. Highly dispersed and stable Schiff base nickel catalyst on multi-walled carbon nanotubes promote ethylene oligomerization. *Chem. Eng. J.* **2024**, 497. DOI: 10.1016/j.cej.2024.154447.

(9) Ngcobo, M.; Ouissa, A.; Kleist, W.; Thiel, W. R.; Ojwach, S. O. Regulating the physical properties of silica immobilized Fe(II), Ni(II) and Co(II) catalysts towards ethylene oligomerization reactions. *Mol. Catal.* **2023**, 549. DOI: 10.1016/j.mcat.2023.113465.
